# Transcriptome profiling in preadipocytes identifies long noncoding RNAs as Sam68 targets

**DOI:** 10.18632/oncotarget.17813

**Published:** 2017-05-11

**Authors:** Naomi Li, Steven Hébert, Jingwen Song, Claudia L. Kleinman, Stéphane Richard

**Affiliations:** ^1^ Segal Cancer Center, Sir Mortimer B Davis Jewish General Hospital, Lady Davis Institute for Medical Research, Montréal, Québec H3T 1E2, Canada; ^2^ Department of Medicine, McGill University, Montréal, Québec H3A 1A1, Canada; ^3^ Department of Oncology, McGill University, Montréal, Québec H3A 1A1, Canada; ^4^ Department of Human Genetics, McGill University, Montréal, Québec H3A 1B1, Canada

**Keywords:** Sam68, RNA binding proteins, lncRNAs, preadipocytes, adipogenesis

## Abstract

The KH-type RNA binding protein Sam68 is required for adipogenesis. We have previously shown that Sam68-deficient mice have a lean phenotype and are protected against dietary-induced obesity due to defects in mTOR and S6K1 alternative splicing. Herein we profiled the transcriptome of Sam68 wild type and deficient 3T3-L1 mouse preadipocytes. We identified 652 protein-coding genes and 9 ncRNAs that were significantly altered with the loss of Sam68. As expected, downregulated genes were significantly associated with GO terms linked to cell migration, motility, and fat cell differentiation, while upregulated genes were mostly associated with GO terms linked to neurogenesis. Of the lncRNAs, we identified *Hotair*, *Mir155hg*, as well as two new lncRNAs (*SR-lncRNA-1* and *SR-lncRNA-2*) that were regulated by Sam68, and contained consensus Sam68 binding sites. RNA stability assays showed that Sam68-deficiency decreased the half-life of *Hotair,* and increased the half-lives of *Mir155hg* and *SR-lncRNA-2*, while the stability of *SR-lncRNA-1* was unaffected. Depletion of *Hotair* and *SR-lncRNA-1* in wild type 3T3-L1 cells led to defects in adipogenesis, whereas depletion of *SR-lncRNA-2* in Sam68-deficient 3T3-L1 cells partially rescued the adipogenesis defect observed in these cells. Collectively, our findings define a new role for Sam68 as a regulator of lncRNAs during adipogenic differentiation.

## INTRODUCTION

With the soaring prevalence in obesity and diabetes, understanding the molecular mechanisms of adipogenesis has become an area of immense interest, and much of our current knowledge has emphasized on the transcriptional cascade that governs adipocyte differentiation [1]. Specifically, the peroxisome proliferator-activated receptor γ (PPARγ) is an essential adipogenic transcription factor, which cooperates with CCAAT/enhancer-binding proteins (C/EBPs), mainly C/EBPα, to induce the expression of genes important for differentiation [1].

The Src-associated in mitosis of 68kDa (Sam68) is an RNA binding protein belonging to the signal transduction and activation of RNA (STAR) family [2–4]. We have previously identified Sam68 as a critical regulator of adipogenesis, where whole-body Sam68 knockout (*Sam68*^*-/-*^) mice are lean due to increased energy expenditure, decreased commitment to early adipocyte progenitors, and defects in adipogenic differentiation [5]. Consistently, mouse embryonic fibroblasts (MEFs) isolated from *Sam68*^*-/-*^ mice are compromised in their ability to differentiate into adipocytes [6]. More importantly, similar to the phenotype found in adipose tissue-specific PPARγ knockout mice [7], *Sam68*^*-/-*^ mice are protected against dietary-induced obesity, as well as insulin resistance and glucose intolerance associated with diabetes [5]. Molecular evidence to date demonstrates that Sam68 can promote adipogenesis by regulating the alternative splicing of mTOR and S6K1 transcripts [5, 8]. Specifically, Sam68-depletion leads to decreased intron 5 retention of the mTOR pre-mRNA, which reduces mTOR protein levels, and causes defects in insulin-stimulated S6 and Akt phosphorylation [5]. In contrast, Sam68 prevents the production of adipogenic repressor p31S6K1, which is encoded by the short isoform of S6K1 [8]. However, it is unclear whether Sam68 has functions beyond that of a splicing regulator during adipogenesis. Moreover, although Sam68-deficient preadipocytes are defective in differentiation, little is known about the exact role of Sam68 in these cells.

A substantial proportion of the genome is transcribed into long noncoding RNAs (lncRNAs), which are RNA transcripts longer than 200 nucleotides, often polyadenylated, and lack evident open reading frames [9]. Despite their biochemical resemblance to messenger RNAs (mRNAs), lncRNAs are poorly conserved, lower in abundance, and have greater tissue specificity [9, 10]. Nevertheless, mounting evidence suggests that specific lncRNAs have regulatory roles in numerous cellular processes, including cell differentiation, development, and disease pathogenesis [9–12]. Previous large-scale studies have shown that lncRNAs can influence adipogenic differentiation, most of which are expressed in a tissue-specific manner, and are strongly induced during adipogenesis [13, 14]. Furthermore, results obtained from gain and loss-of-function screens suggest that some lncRNAs can serve as key regulators of adipocyte-specific genes [13, 15]. Indeed, a few lncRNAs have been characterized with important roles in adipocyte development, including *ADINR* [15], *HOTAIR* [16], *NEAT1* [17, 18], as well as *lnc-BATE-1* specifically for brown adipocyte differentiation [14]; however, the function and regulation for many existing lncRNAs remain poorly understood.

In the present study, we performed deep RNA sequencing (RNA-seq) to profile the transcriptome influenced by the loss of Sam68 in mouse preadipocytes. We found a small subset of lncRNAs that are differentially regulated by Sam68, which contained consensus Sam68 binding sites, and are modulated by Sam68 via transcript stability. Furthermore, we confirmed by RNA interference (RNAi) that these lncRNAs play an important role in preadipocyte differentiation. Hence, we have characterized a new role for Sam68 as a regulator of lncRNAs during adipogenesis.

## RESULTS

### Transcriptional profiling of Sam68 wild type and deficient preadipocytes

**Figure 1:**
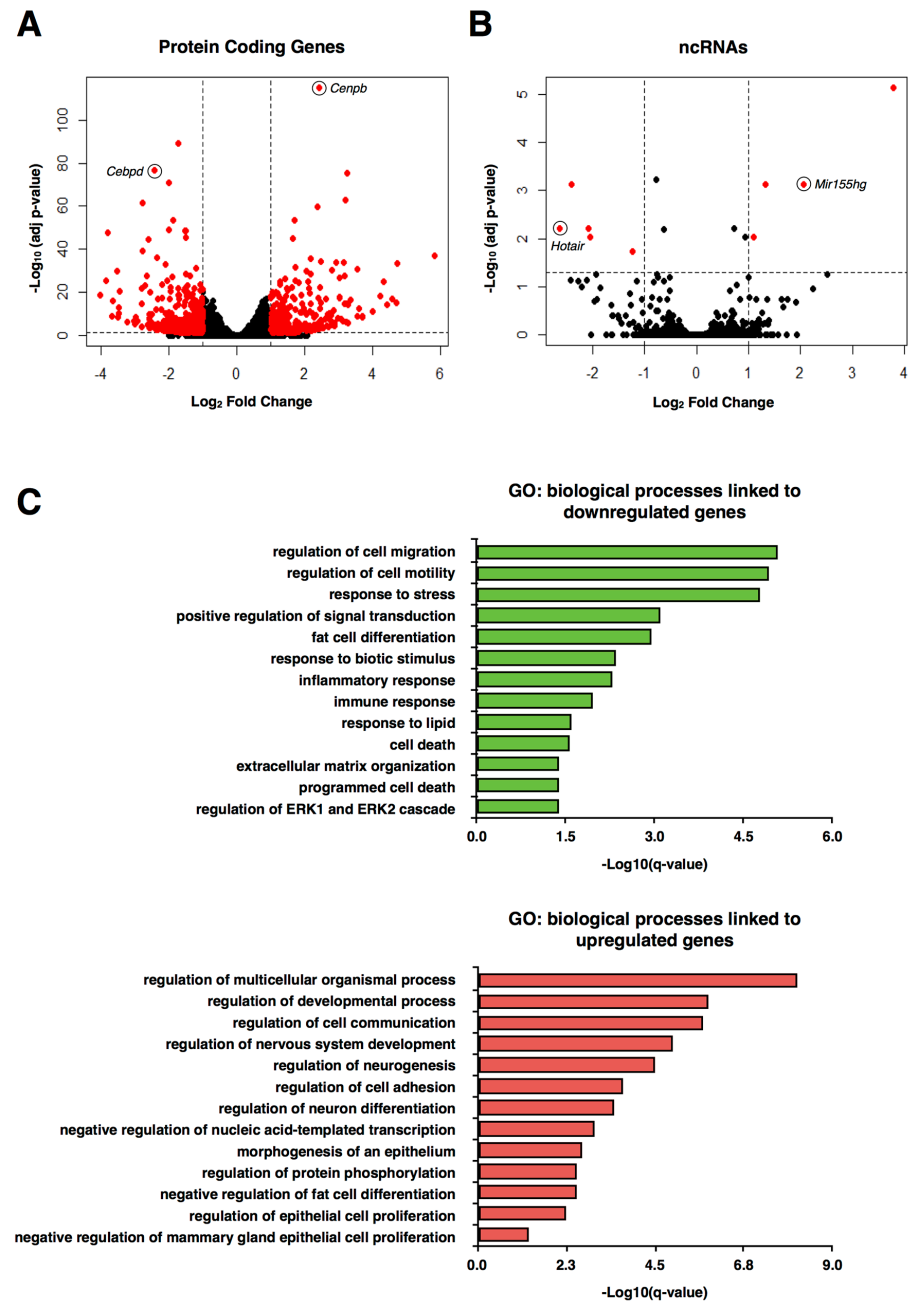

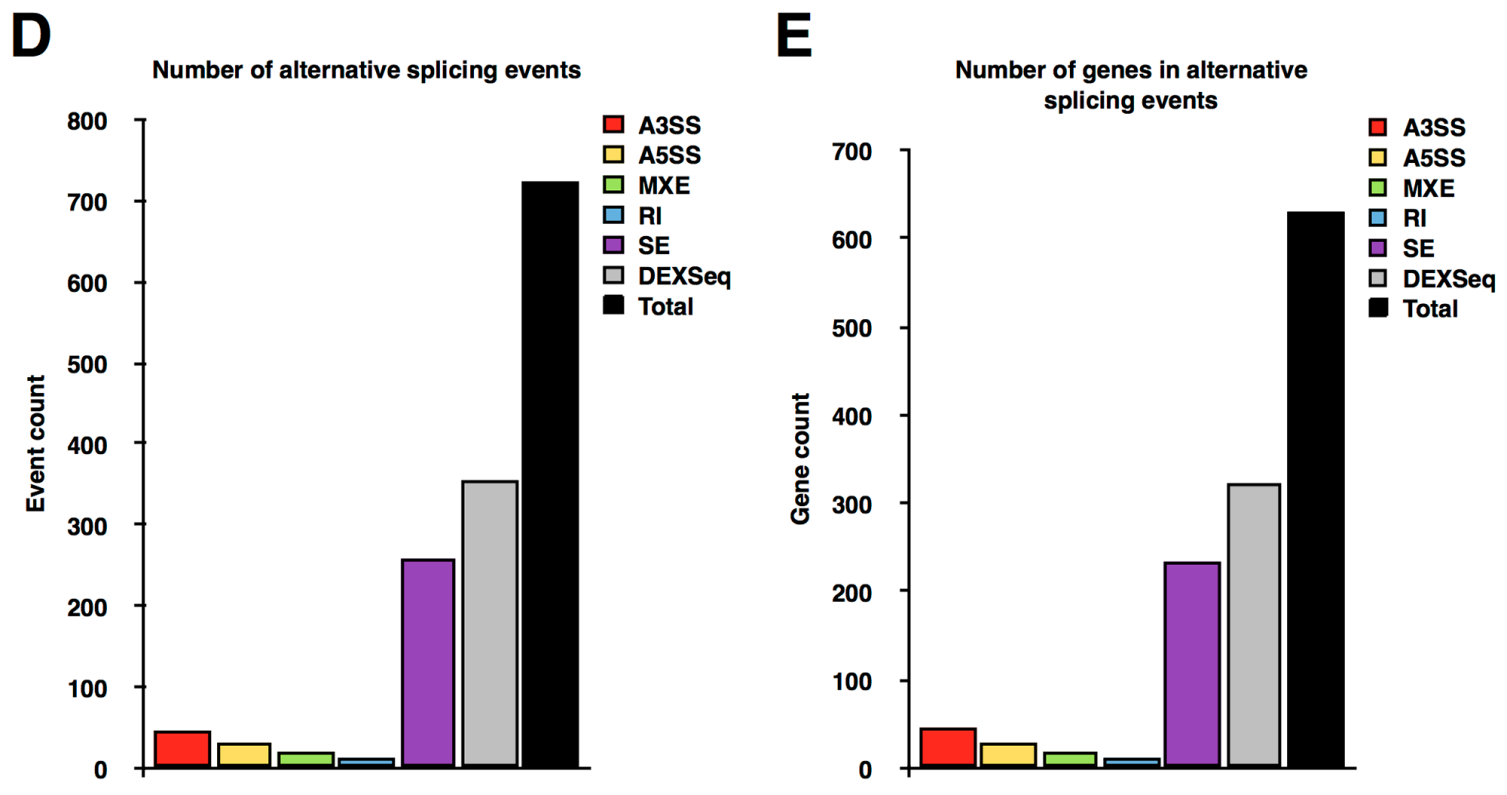
RNA-seq analysis of protein-coding genes and ncRNAs in mouse preadipocytes (**A** and **B**) Volcano plot of protein-coding genes (A) and noncoding RNAs (ncRNAs) (B) differentially expressed between pRetroSuper and Sam68sh 3T3-L1 cells (red). (**C**) Most significant gene ontology (GO) terms associated with downregulated (green) and upregulated (red) genes in Sam68sh 3T3-L1 cells. **(D** and **E)** Differential alternative splicing events detected by rMATS and DEXSeq between pRetroSuper and Sam68sh 3T3-L1 preadipocytes. Bar plots represent the number of each type of alternative splice event (D) and the number of different genes found in each event (E). A3SS, alternative 3’ splice site; A5SS, alternative 5’ splice site; MXE, mutually exclusive exons; RI, retained intron; SE, skipped exon.

To identify the transcripts whose expression is regulated by Sam68, we first performed RNA-seq using undifferentiated Sam68 wild type (pRetroSuper) and Sam68-deficient (Sam68sh) 3T3-L1 cells. These Sam68sh cells were previously characterized for their differentiation defects [5]. We identified 652 protein-coding genes and 9 noncoding RNAs (ncRNAs) that were significantly up or downregulated (≥ 2 fold change, adjusted *p* < 0.05) by Sam68 (Figures 1A and 1B, Supplemental Tables 1 and 2). Indeed, the expression of pro-adipogenic factors such as C/EBP δ (*Cebpd*) (adjusted *p* = 1.52 × 10^-77^) was among the most significantly downregulated genes in Sam68sh 3T3-L1 cells, while the gene expression of repressors, for instance GATA-3 (*Gata3*) (adjusted *p* = 1.45 × 10^-5^), was significantly upregulated with Sam68 depletion. And interestingly, the lncRNA *Hotair* (adjusted *p* = 6.14 × 10^-3^) was one of the most downregulated ncRNAs in Sam68-deficient cells. As expected, global gene ontology (GO) analysis revealed that downregulated genes in Sam68-deficient cells were significantly associated with “regulation of cell migration”, “regulation of cell motility”, “fat cell differentiation”, “response to lipid”, “extracellular matrix organization”, and other known Sam68-regulated biological processes (*q* < 0.05) (Figure 1C). Conversely, upregulated genes in Sam68-deficient cells were significantly associated with GO terms linked to “regulation of neurogenesis”, “negative regulation of fat cell differentiation”, and “negative regulation of nucleic acid-templated transcription” (*q* < 0.05, Figure 1C), all of which are consistent with previous reports of Sam68 functions [19–21]. Since Sam68 is known to regulate alternative splicing during adipogenesis [5, 8], we also analyzed the data for genome-wide alternation in transcript usage. Indeed, we detected a total of 725 differential alternative splicing events across 631 genes between wild type and Sam68-deficient 3T3-L1 preadipocytes (Figures 1D and 1E, Supplemental Table 3). We did not detect the previously identified *mTOR* and *Rps6kb1* alternative splicing events, likely because of the limited sequence coverage. Further analysis will be required to confirm the new alternative spliced events identified.

### Genome-wide identification of Sam68-regulated lncRNAs in preadipocytes

**Figure 2:**
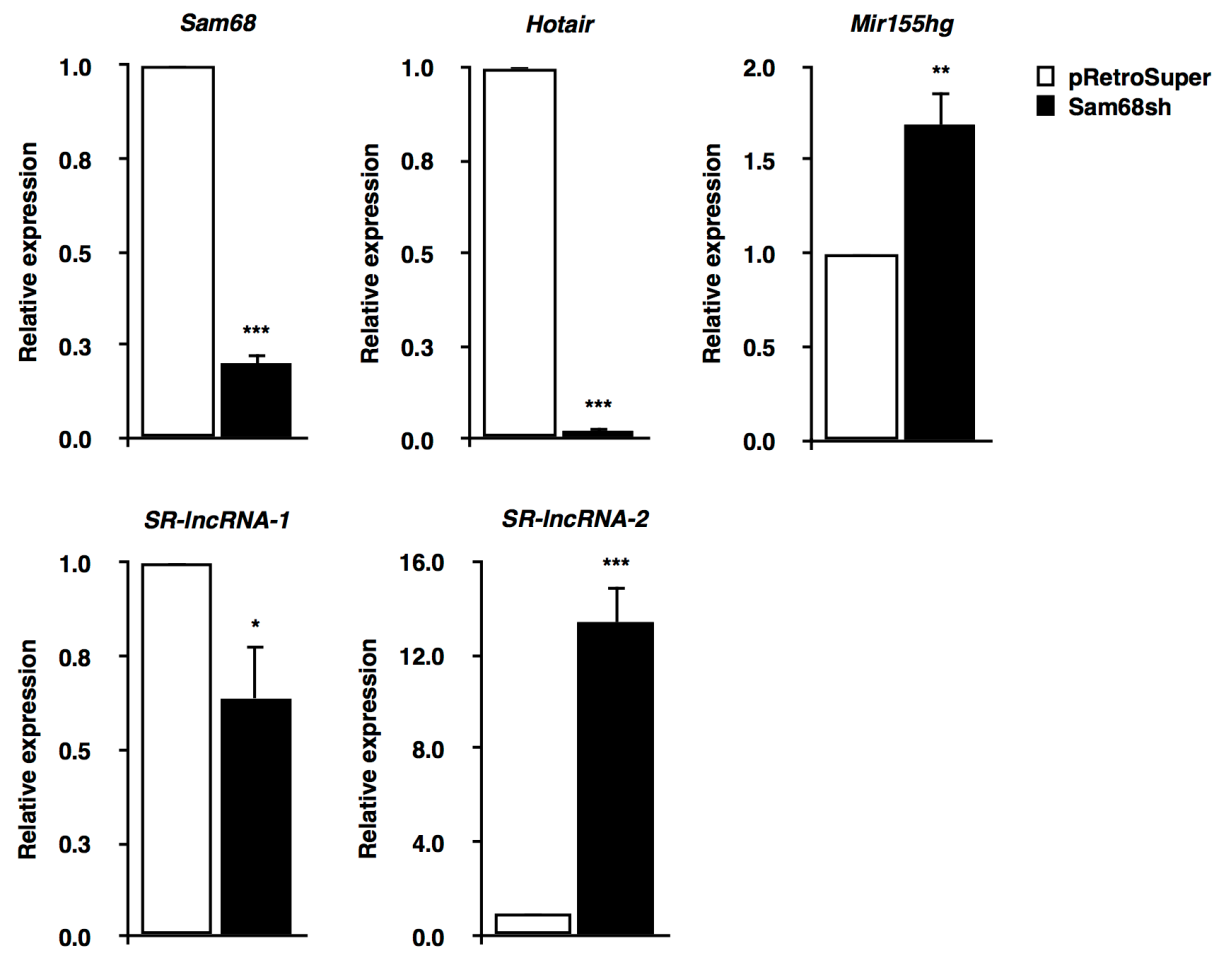
Validation of Sam68 lncRNA targets RT-qPCR confirmation of *Sam68* mRNA expression and Sam68-regulated lncRNAs (*Hotair*, *Mir155hg*, *SR-lncRNA-1*, *SR-lncRNA-2*) in pRetroSuper (white) and Sam68sh (black) 3T3-L1 cells. Expressions were normalized to 18S rRNA levels. Data are represented as mean ± S.D. from 3 independent experiments done in biological triplicates (*p ≤0.05, **p ≤0.005, ***p ≤0.0005).

Detailed analysis of the 9 ncRNAs identified from our RNA-seq data revealed lncRNAs, microRNAs (miRNAs), small cytoplasmic RNAs (scRNAs), and uncharacterized RIKEN sequences that were differentially regulated by Sam68. To further investigate the role of Sam68 in lncRNA regulation, we performed RT-qPCR in pRetroSuper and Sam68sh 3T3-L1 cells. We confirmed the depletion of *Sam68* mRNA (∼80%), and we validated 4 lncRNAs that were differentially regulated by Sam68 (Figure 2). These lncRNAs include 2 known transcripts, *Hotair* and *Mir155hg*, as well as 2 previously uncharacterized lncRNAs herein referred to as Sam68-regulated lncRNAs (*SR-lncRNAs*), *SR-lncRNA-1* (2610035D17Rik) and *SR-lncRNA-2* (4930461G14Rik). Our results showed that Sam68-deficiency significantly decreased *Hotair* (∼50-fold) and *SR-lncRNA-1* (∼1.7-fold) levels, while the expression of *Mir155hg* and *SR-lncRNA-2* were substantially increased by ∼1.7-fold and ∼13-fold, respectively, compared to Sam68 wild type cells (Figure 2). Of note, ectopic expression of *Hotair* has been shown to promote preadipocyte differentiation [16], whereas the mature product of *Mir155hg*, miR-155, is a known inhibitor of adipogenesis [22]. Together, we identify lncRNA targets for Sam68 in preadipocytes that are both positive and negative regulators of adipogenesis.

### Sam68 controls lncRNA expression by regulating transcript stability

To understand how Sam68 regulates the expression of lncRNAs in preadipocytes, we first searched for potential Sam68 binding sites in the available sequences for *Hotair*, *Mir155hg*, *SR-lncRNA-1*, and *SR-lncRNA-2*, given that Sam68 binds to UU/AAA bipartite elements [23, 24]. While we found potential Sam68 binding sites on all 4 lncRNAs (Table 1), only *Hotair*, *Mir155hg*, and *SR-lncRNA-2* contained perfect consensus Sam68 binding sites.

**Table 1:** Consensus Sam68 binding sites on target lncRNAs.

Target	RefSeq	Consensus Sam68 binding sites	Nucleotides (bp)
***Hotair***	NR_047528.1	…**UUAA**GCTG**UAAA**…**UUUA**UUUU**UAAA**…	26-37, 1916-1927
***Mir155hg***	NR_132106.1	…**UAAA**UAU**UUAA**U**UUAAUUAA**UAU**UUAA**…	1032-1058
***SR-lncRNA-1***	NR_015556.1	…**UUUA**UA**UAAA**…**UAAA**UUCC**UUUA**…	1605-1614, 1834-1845
***SR-lncRNA-2***	NR_040736.1	…**UUAA**AA**UUAA**…	731-740

**Figure 3:**
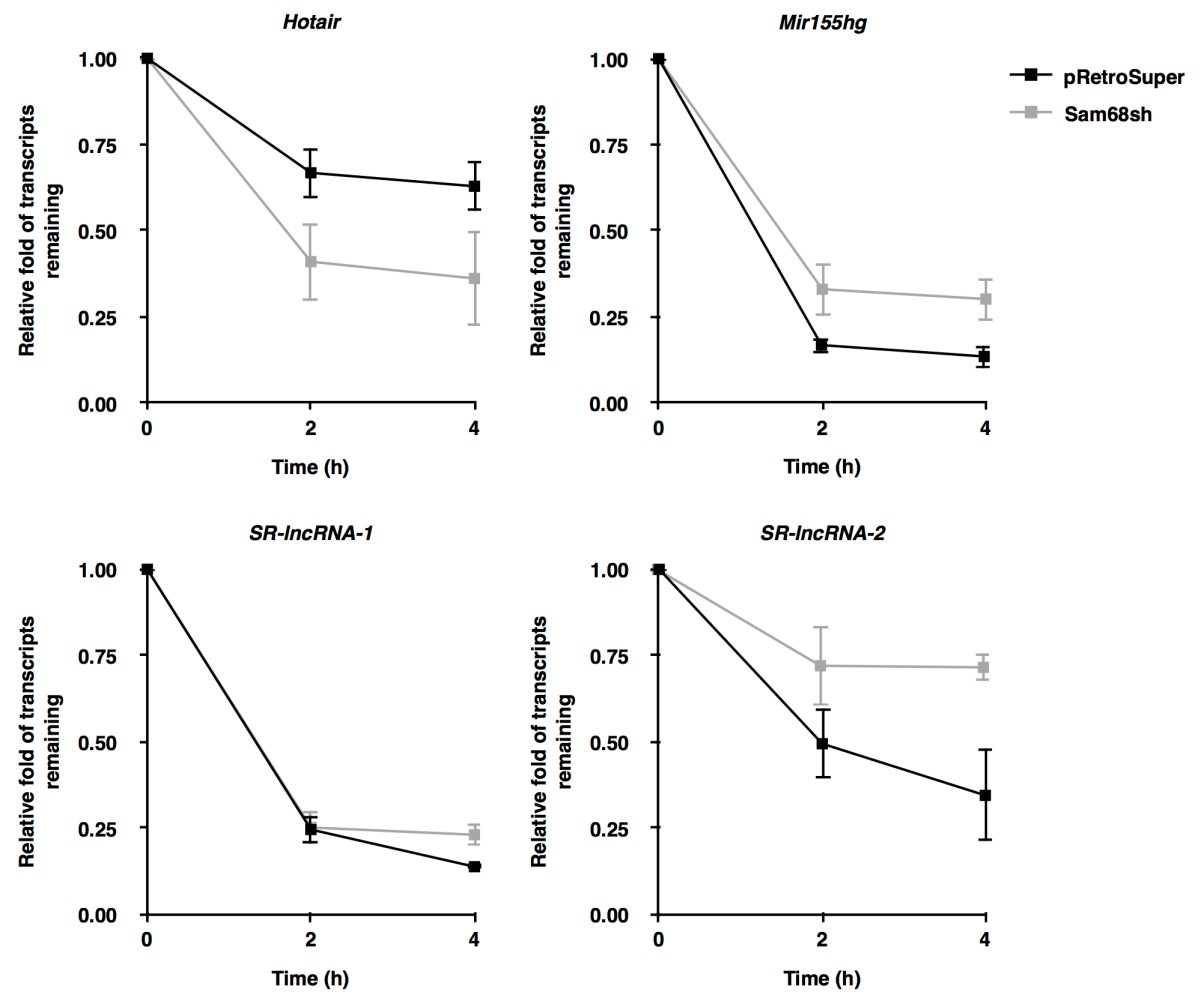
Sam68 regulates lncRNA expression by modulating transcript stability Transcript stability was assessed in pRetroSuper (black) or Sam68sh (grey) 3T3-L1 cells treated with actinomycin D (5μg /mL). Total RNA was extracted at the indicated time points and transcripts were quantified by RT-qPCR using primers specific for *Hotair*, *Mir155hg*, *SR-lncRNA-1*, and *SR-lncRNA-2*, and were normalized to 18S rRNA levels. Data are represented as mean ± S.D. from 3 independent experiments done in biological triplicates.

We next performed actinomycin D experiments to monitor the half-life of *Hotair*, *Mir155hg*, *SR-lncRNA-1*, and *SR-lncRNA-2* in pRetroSuper and Sam68sh 3T3-L1 cells. Our results demonstrate that Sam68 status affects the transcript half-life of 3 out of the 4 lncRNAs (Figure 3). Specifically, while depletion of Sam68 did not significantly influence the half-life of *SR-lncRNA-1* (1.98 h vs. 1.91 h), which does not contain a perfect Sam68 binding site, the half-life of *Hotair* was dramatically reduced from 3.43 h to 2.28 h (Figure 3). In contrast, Sam68-deficiency significantly increased the transcript half-life of *Mir155hg* (1.85 h vs. 2.13 h) and *SR-lncRNA-2* (2.35 h vs. 4.19 h) (Figure 3). Altogether, these data suggest that Sam68 mainly modulates target lncRNA expression by differentially regulating their transcript stability. However, given the evidence of Sam68 as a transcriptional regulator [25–27], we do not exclude the possibility that Sam68 may also contribute to lncRNA expression in preadipocytes at the transcription level.

### Sam68-regulated lncRNAs in preadipocytes are important for adipogenic differentiation

**Figure 4:**
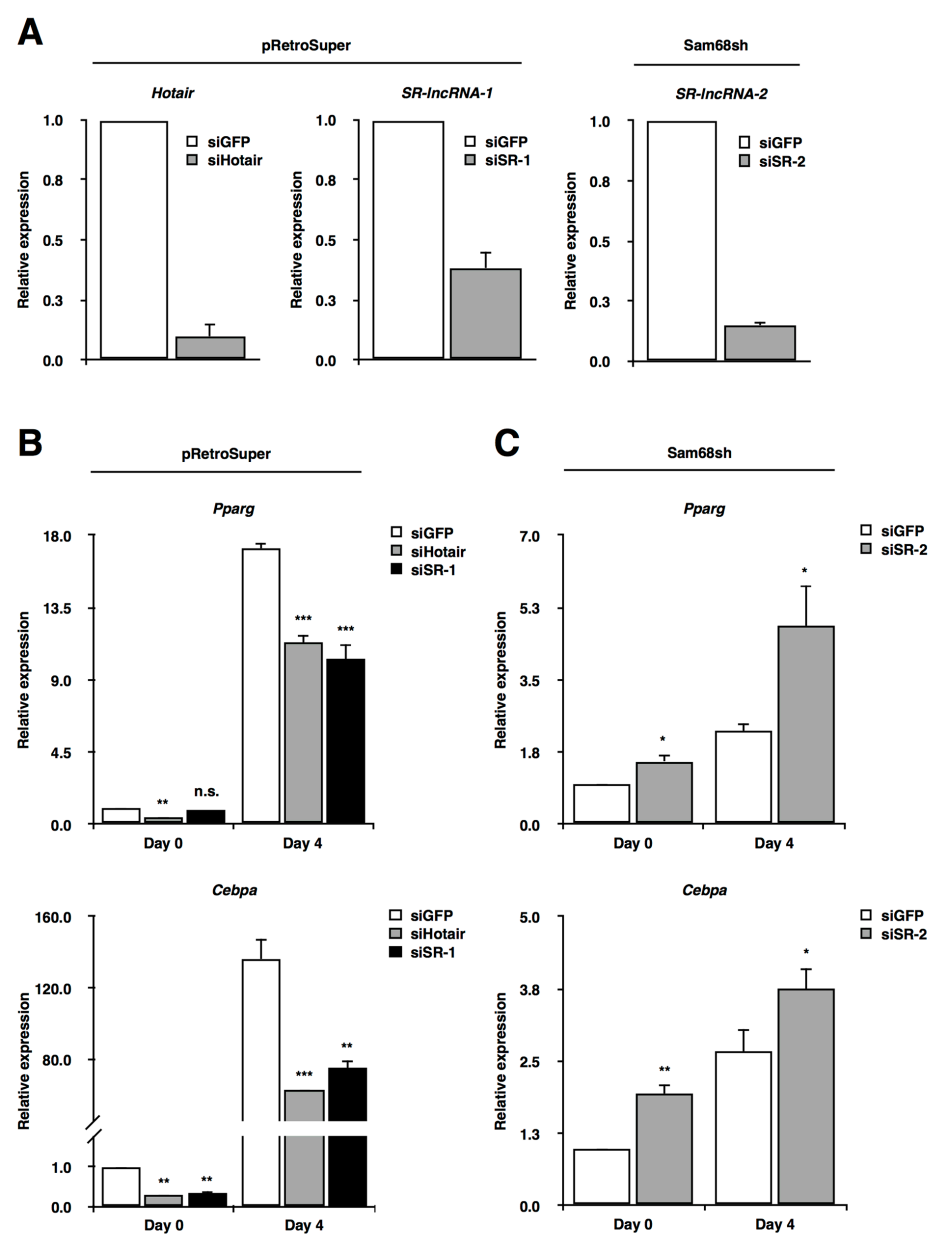
Sam68-regulated lncRNAs are required for adipogenic differentiation **(A)** RT-qPCR confirmation of lncRNA knockdown in pRetroSuper (*Hotair*, *SR-lncRNA-1*) and Sam68sh (*SR-lncRNA-2*) 3T3-L1 cells. Expressions were normalized to 18S rRNA levels. **(B** and **C)** RT-qPCR analysis of *Pparg* and *Cebpa* on day 0 and day 4 of adipogenic differentiation after knockdown of the indicated lncRNAs in pRetroSuper (B) and Sam68sh 3T3-L1 cells (C). Expressions were normalized to 18S rRNA levels. Data, replicated in 3 independent experiments, are represented as mean ± S.D. of biological triplicates (*p ≤0.05, **p ≤0.005, ***p ≤0.0005).

To assess the functional role of Sam68-regulated lncRNAs in adipogenesis, we next performed loss-of-function studies using small interfering RNAs (siRNAs). Our earlier data showed that *Hotair* and *SR-lncRNA-1* had a higher expression in wild type 3T3-L1 cells compared to Sam68sh cells, whereas it was the contrary for *SR-lncRNA-2*. Thus, we decided to deplete *Hotair* and *SR-lncRNA-1* transcripts from wild type 3T3-L1 cells, and *SR-lncRNA-2* from Sam68sh 3T3-L1 cells (Figure 4A).

Consistent with previous studies demonstrating *Hotair* as a positive regulator of adipogenesis [16], knockdown of *Hotair* markedly reduced the expression of adipogenic marker genes *Pparg* and *Cebpa* on both day 0 and day 4 of differentiation (Figure 4B). Similarly, although knockdown of *SR-lncRNA-1* did not significantly alter *Pparg* mRNA levels on day 0, *Pparg* induction was compromised on day 4 compared to control cells, while *Cebpa* expression was dramatically decreased on both day 0 and day 4 with *SR-lncRNA-1* knockdown compared to control cells (Figure 4B). Unlike *Hotair* and *SR-lncRNA-1*, Sam68 depletion increased *SR-lncRNA-2* expression (Figure 2), suggesting that *SR-lncRNA-2* may be an early inhibitor of adipogenic differentiation. Indeed, although not comparable to Sam68 wild type cells, depletion of *SR-lncRNA-2* in Sam68sh cells significantly increased *Pparg* and *Cebp*a mRNA levels on day 0 and day 4 of differentiation compared to Sam68sh control cells (Figure 4C).

**Figure 5:**
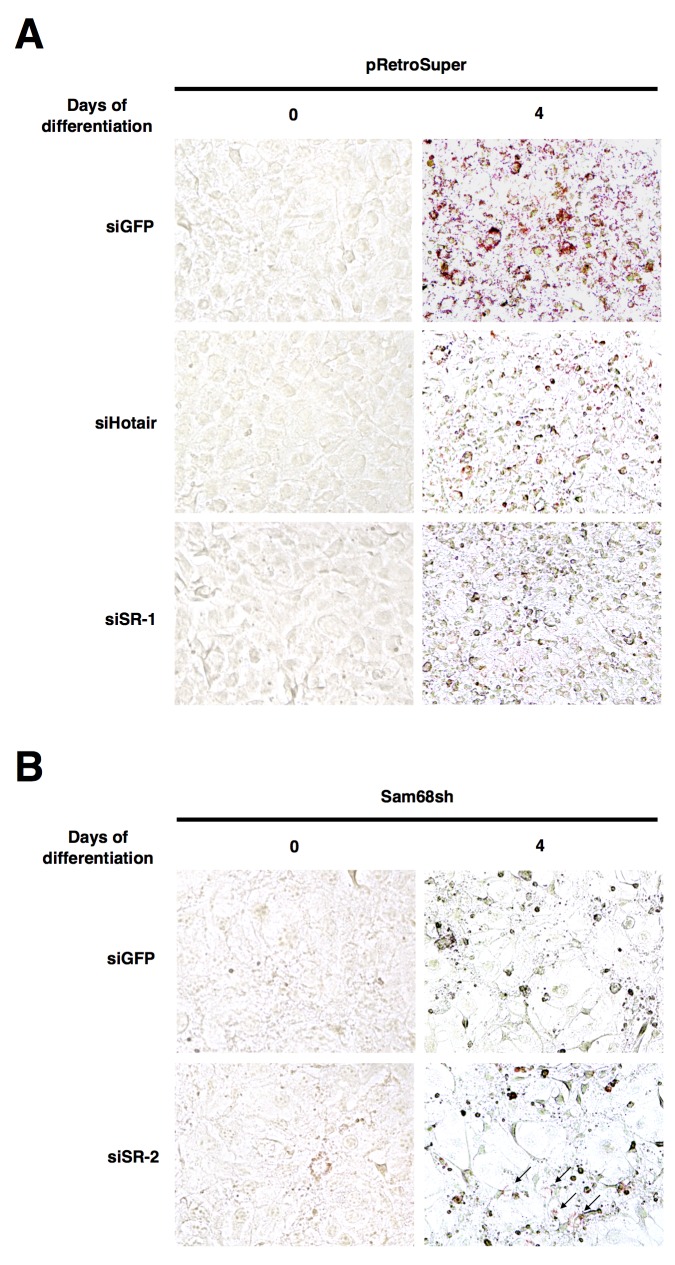
Sam68-regulated lncRNAs are required for lipid accumulation **(A** and **B)** Oil red O staining (40X) on day 0 and day 4 of adipogenic differentiation after knockdown of the indicated lncRNAs in pRetroSuper (A) and Sam68sh (B) 3T3-L1 cells.

To confirm our gene expression data, we performed oil red O (ORO) staining for lncRNA-depleted 3T3-L1 cells on day 0 and day 4 of differentiation to monitor lipid accumulation. Consistently, the number of ORO^+^ cells was dramatically reduced when *Hotair* or *SR-lncRNA-1* was depleted, as a result of defective adipogenic differentiation (Figure 5A). On the other hand, knockdown of *SR-lncRNA-2* visibly increased the number of ORO^+^ Sam68sh cells on day 4 of differentiation, suggesting a slight rescue in adipogenic differentiation (Figure 5B). Collectively, these results demonstrate that Sam68-regulated lncRNAs have critical functions in adipogenesis.

## DISCUSSION

Although the study of adipogenesis has traditionally focused on nuclear transcription factors, lncRNAs are recently emerging as key players in adipogenic differentiation [11, 13, 14]. In the present manuscript, we unveiled a new role for Sam68 as a regulator of lncRNAs in mouse preadipocytes to promote adipogenesis. We identified Sam68-regulated lncRNAs that have important functions in adipocyte differentiation including *Hotair*, *Mir155hg*, *SR-lncRNA-1*, and *SR-lncRNA-2*, where the latter two are new lncRNAs that have never been previously associated with adipogenesis. These lncRNAs contain consensus Sam68 binding sites, specifically with the stability of *Hotair*, *Mir155hg*, and *SR-lncRNA-2* modulated by Sam68. These findings define a new role for Sam68 during adipogenesis, namely in its regulation on transcript stability.

LncRNAs are known to regulate adipogenesis through lncRNA-protein interactions [11]; however few studies have linked lncRNAs with RNA binding proteins. Given that Sam68 is required for adipogenesis [5, 8], we now demonstrate its importance in cell differentiation as a regulator of specific lncRNAs using mouse preadipocytes. Interestingly, Sam68 has also been described to modulate lncRNA functions in human cancer cells. Specifically, *INXS* requires the interaction of Sam68-containing ribonucleoprotein complexes for efficient *BCL-XS* splicing, leading to increased apoptosis and tumor regression [28]. Furthermore, Sam68 associates with the p53 lncRNA target, *PR-lncRNA-1*, to synergistically enhance p53 transcriptional activities and effector functions [25]. Taken together, these studies suggest that the role of Sam68 as lncRNA regulator is conserved across different species, cell types, and cellular networks.

Much of the previous studies have focused on identifying differentially expressed lncRNAs during adipocyte differentiation [13, 14]. We now add to these studies by defining new functional lncRNAs in preadipocytes. Among our validated Sam68 lncRNA targets, *Hotair* is one of the few lncRNAs with well-characterized molecular functions. In humans, increased *HOTAIR* expression is associated with promotion of cancer metastasis, where it has been shown to reprogram the cellular chromatin state by re-targeting Polycomb Repressive Complex 2 (PRC2) [29]. Additionally to cancer, *HOTAIR* expression is also detected in gluteal adipose tissues, with enrichment in adipocyte fractions, suggesting its potential role in adipogenic differentiation (Supplemental Figure 1) [16, 30]. Indeed, ectopic expression of *HOTAIR* promotes adipogenesis [16]. We now extend these findings, and show that Sam68 increases *Hotair* stability to promote adipogenesis.

Moreover, we also identified *Mir155hg*, which is the primary sequence of miR-155, a known inhibitor of adipogenesis [22]. Although the relation between Sam68 and miRNAs was not investigated in our current study, many publications have shown that lncRNAs can block miRNA maturation by complementing with their primary sequences, or reduce miRNA levels by acting as sponges [10, 11]. In addition to the two annotated targets, we discovered two previously uncharacterized Sam68-regulated lncRNAs, *SR-lncRNA-1* and *SR-lncRNA-2*, with positive and negative roles in adipocyte differentiation, respectively. Given the versatility and ubiquitous nature of Sam68 [3, 4], it would be of great interest to further explore their possible functions in other cellular processes and diseases.

One aspect of Sam68 that we have yet to investigate is its possible transcriptional role in preadipocytes. Indeed, previous studies have identified Sam68 as a co-regulator for a number of transcription factors, including the androgen receptor (AR) [27], NF-κB [26], and p53 [25]. These evidences suggest that Sam68 may contribute to the preadipocyte transcriptome by modulating both the transcription and transcript stability of its target genes. Moreover, it is interesting to note that Sam68 can differentially regulate transcript stability. Specifically, while Sam68-deficiency decreased the half-life of *Hotair*, it increased the half-lives of *Mir155hg* and *SR-lncRNA-2*. Given that spliced lncRNAs are more stable than single exon transcripts [31], it can be speculated that Sam68 may influence stability by regulating the splicing of its target lncRNAs.

To date, several mechanisms of action have been elucidated for lncRNAs in gene regulation. Most lncRNAs are nuclear, and function by modifying chromatin structures [32, 33], where they act as molecular scaffolds to recruit DNA methyltransferases [34], or histone modification complexes that ultimately influence transcription [35]. On the other hand, cytoplasmic lncRNAs can modulate translation, regulate mRNA degradation, or act as sponges for miRNAs [36].

In summary, our study demonstrates a previously unappreciated role for Sam68 as a regulator of lncRNAs during adipogenesis. This feature of Sam68 can be potentially harnessed as a therapeutic target for treating obesity and its related metabolic disorders, possibly by inhibiting the activity of Sam68 [37]. However, much remains to be determined on deciphering the detailed mechanisms by which Sam68 acts through lncRNAs for regulating gene expression.

## MATERIALS AND METHODS

### RNA-seq, GO, and alternative splicing event analysis

Total RNA was isolated from undifferentiated Sam68 wild type (pRetroSuper) and Sam68-deficient (Sam68sh) 3T3-L1 preadipocytes using TRIzol reagent according to the manufacturer’s instructions. RNA integrity and quality were checked as previously described [5], and biological triplicates of each sample were sent for sequencing. For analysis, raw reads were trimmed using Trimmomatic v0.32 [38], removing low-quality bases at the end of the reads (phred33 < 30), clipping the first 4 bases and clipping the Illumina adaptor sequences using palindrome mode. A sliding window quality trimming was performed, cutting once the average quality of a window of 4 bases that fell below 30. Reads shorter than 30 base pairs after trimming were discarded. Trimmed reads were aligned to the reference genome mm10 using STAR v2.3.0e [39]. Read counts for each gene at the exonic level were calculated by featureCounts v1.4.4 using Gencode VM7 annotation, normalized using DESeq2 R library [40], and were annotated using org.Mm.eg.db R bioconductor library. Differential expression analysis was performed using DESeq2 for all Gencode VM7 genes at the exonic level [40]. Genes were considered differentially expressed if they had a |fold change| ≥ 2 and an adjusted *p*-value < 0.05. Gene ontology (GO) analysis was performed with GOrilla tools [41]. Differential alternative splicing events were detected using rMATS (FDR < 0.05) and DEXSeq (adjusted *p* < 0.05) [42, 43].

### Preadipocyte differentiation and ORO staining

Sam68 wild type (pRetroSuper) and deficient (Sam68sh) 3T3-L1 cells were previously described [5]. Adipogenic differentiation of 3T3-L1 cells was performed as previously described [5]. For oil red O (ORO) staining, cells were fixed in 3% formaldehyde and 0.025% glutaraldehyde. After fixation, cells were washed with PBS and were stained with freshly prepared ORO solution (Sigma-Aldrich).

### RNA extraction and RT-qPCR

Total RNA was extracted at the indicated time points as described above. Reverse transcription (RT) was performed with the M-MLV reverse transcriptase (Promega). Real-time PCR (qPCR), including primer design and efficiency tests, was performed according to the Minimum Information for Publication of Quantitative Real-Time PCR Experiments (MIQE) guidelines [44]. qPCR experiments were performed using PowerUp SYBR Green Master Mix (Thermo Fisher Scientific) following the manufacturer’s instructions. Data were normalized by the ΔΔCt method. Sequences of primers used for qPCR are listed in Table 2.

**Table 2:** List of primers used for qPCR.

Target (mouse)	Forward Primer (5’-3’)	Reverse Primer (5’-3’)
***Sam68***	GTGGAGACCCCAAATATGCCCA	AAACTGCTCCTGACAGATATCA
***Pparg***	GAACGTGAAGCCCATCGAGGAC	CTGGAGCACCTTGGCGAACA
***Cebpa***	CGCAAGAGCCGAGATAAAGC	GCGGTCATTGTCACTGGTCA
***Hotair***	AAGGCTGAAATGGAGGACCG	TACCGATGTTGGGGACCTCT
***Mir155hg***	GGGGTTTTGGCCTCTGACTG	TTGGACTTGTCATCCTCCCAC
***SR-lncRNA-1***	TCCTTGCCCGACTACAAACC	TACAGCTCTGCGCCTTTCTT
***SR-lncRNA-2***	GCATCACTGTGGTCTCTACCC	TAACCCTTCTGCCAAGCTCTC
***18S***	GTAACCCGTTGAACCCCATT	CCATCCAATCGGTAGTAGCG
***Hprt***	TTGGGCTTACCTCACTGCTT	TCGCTAATCACGACGCTGG
***Arp***	GAGGAATCAGATGAGGATATGGGA	AAGCAGGCTGACTTGGTTGC

### LncRNA knockdown

The following siGENOME SMARTpool small interfering RNAs (siRNAs) were purchased from Dharmacon: *Hotair*, *SR-lncRNA-1* (2610035D17Rik), and *SR-lncRNA-2* (4930461G14Rik). Transfection of siRNAs was performed with RNAiMAX reagents (Invitrogen) according to the manufacturer’s instructions. Cells were recovered and grown to confluence before induction of differentiation. Total RNA was harvested as described above.

## SUPPLEMENTARY MATERIALS FIGURE AND TABLES








