# Supplementary material for: Transcriptome profiling in preadipocytes identifies long noncoding RNAs as Sam68 targets
Source: Oncotarget. 2017 May 11;8(47):81994–2005. doi: 10.18632/oncotarget.17813 (PMC5669865; doi:10.18632/oncotarget.17813)
Supplement: Supplementary file 1 [file oncotarget-08-81994-s001.pdf]

Transcriptome profiling in preadipocytes identifies long noncoding RNAs as Sam68 targets

SUPPLEMENTARY MATERIALS

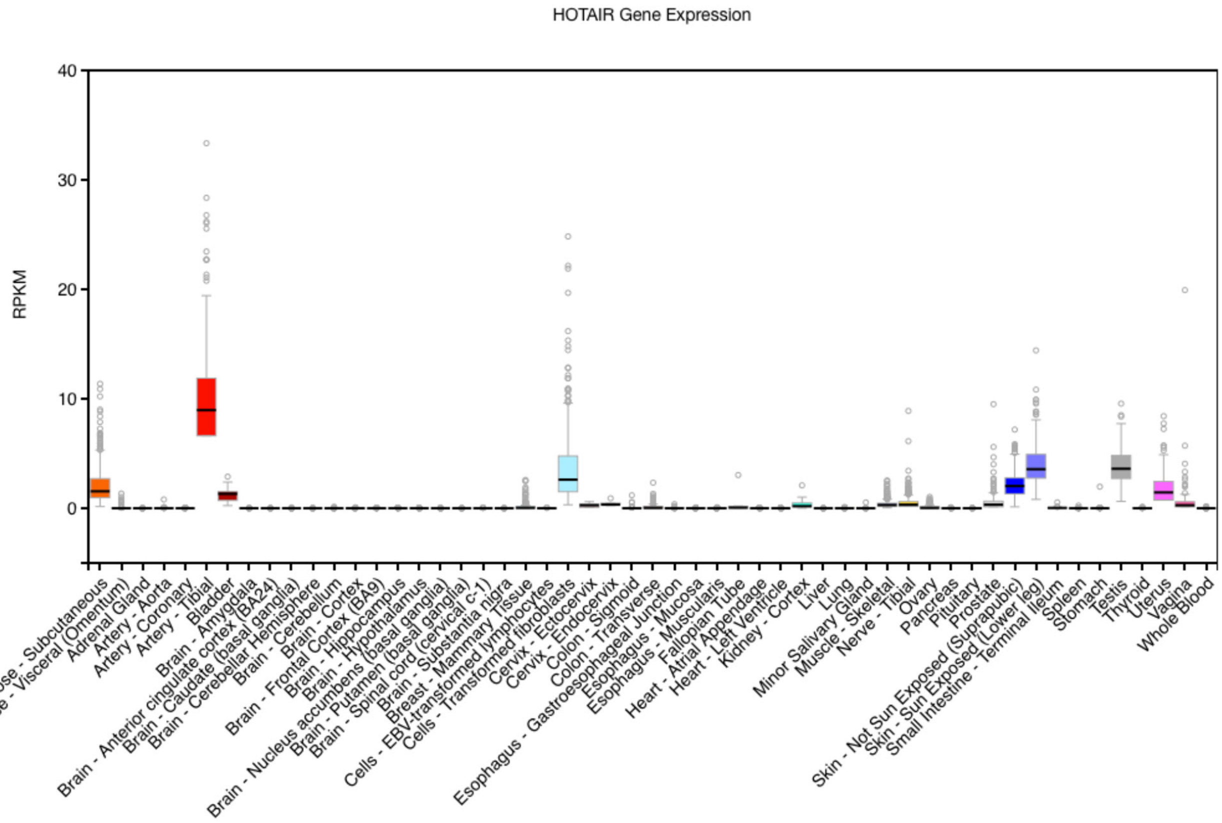

Supplemental : *HOTAIR* expression in various human tissues (GTEx).

Supplemental Table 1: Protein-coding genes up or downregulated by Sam68 in mouse 3T3-L1 preadipocytes.
See Supplementary File 1

Supplemental Table 2: Noncoding RNAs up or downregulated by Sam68 in mouse 3T3-L1 preadipocytes.
See Supplementary File 2

Supplemental Table 3: Alternative splicing events detected by rMATS and DEXSeq in mouse 3T3-L1 preadipocytes.
See Supplementary File 3
